# Supplementary material for: Comparative Meta-Analysis of Triplet vs. Quadruplet Induction Regimens in Newly Diagnosed, Treatment Naïve, Multiple Myeloma
Source: Cancers (Basel). 2024 Aug 23;16(17):2938. doi: 10.3390/cancers16172938 (PMC11394295; doi:10.3390/cancers16172938)
Supplement: Supplementary file 1 [file cancers-16-02938-s001.zip › cancers-3132963-supplementary.pptx]

## Slide 1
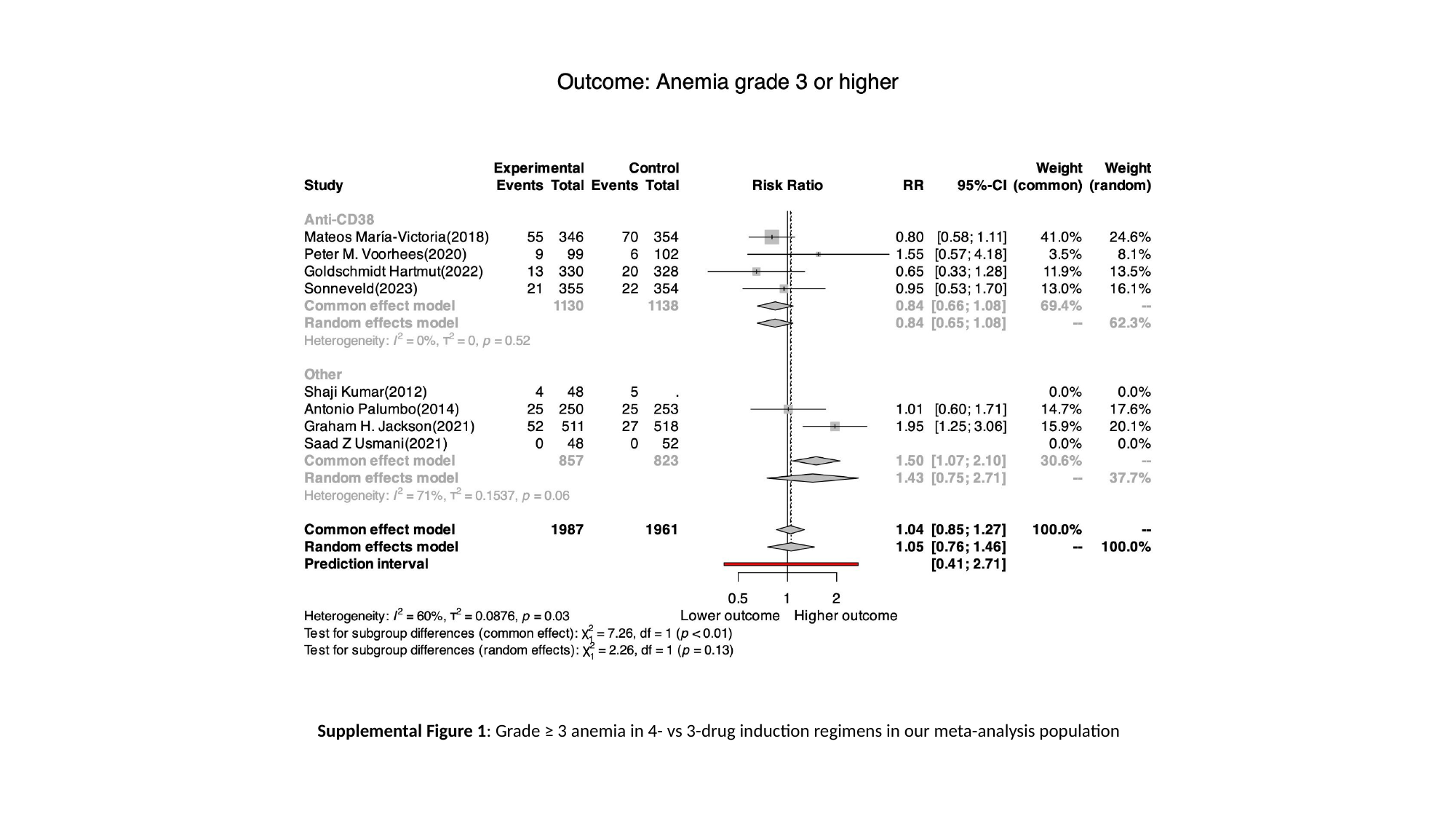

Supplemental Figure 1: Grade ≥ 3 anemia in 4- vs 3-drug induction regimens in our meta-analysis population

## Slide 2
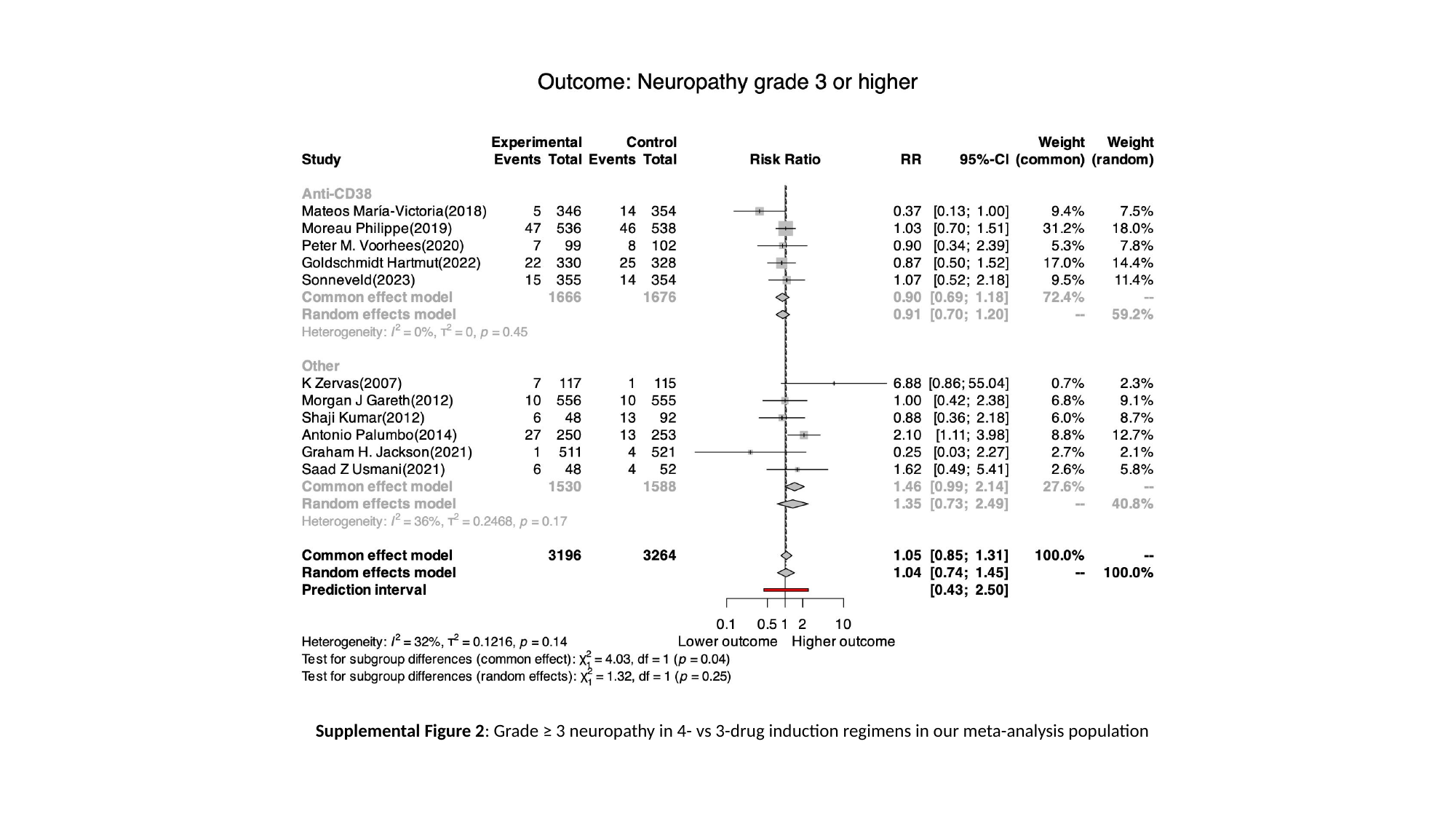

Supplemental Figure 2: Grade ≥ 3 neuropathy in 4- vs 3-drug induction regimens in our meta-analysis population

## Slide 3
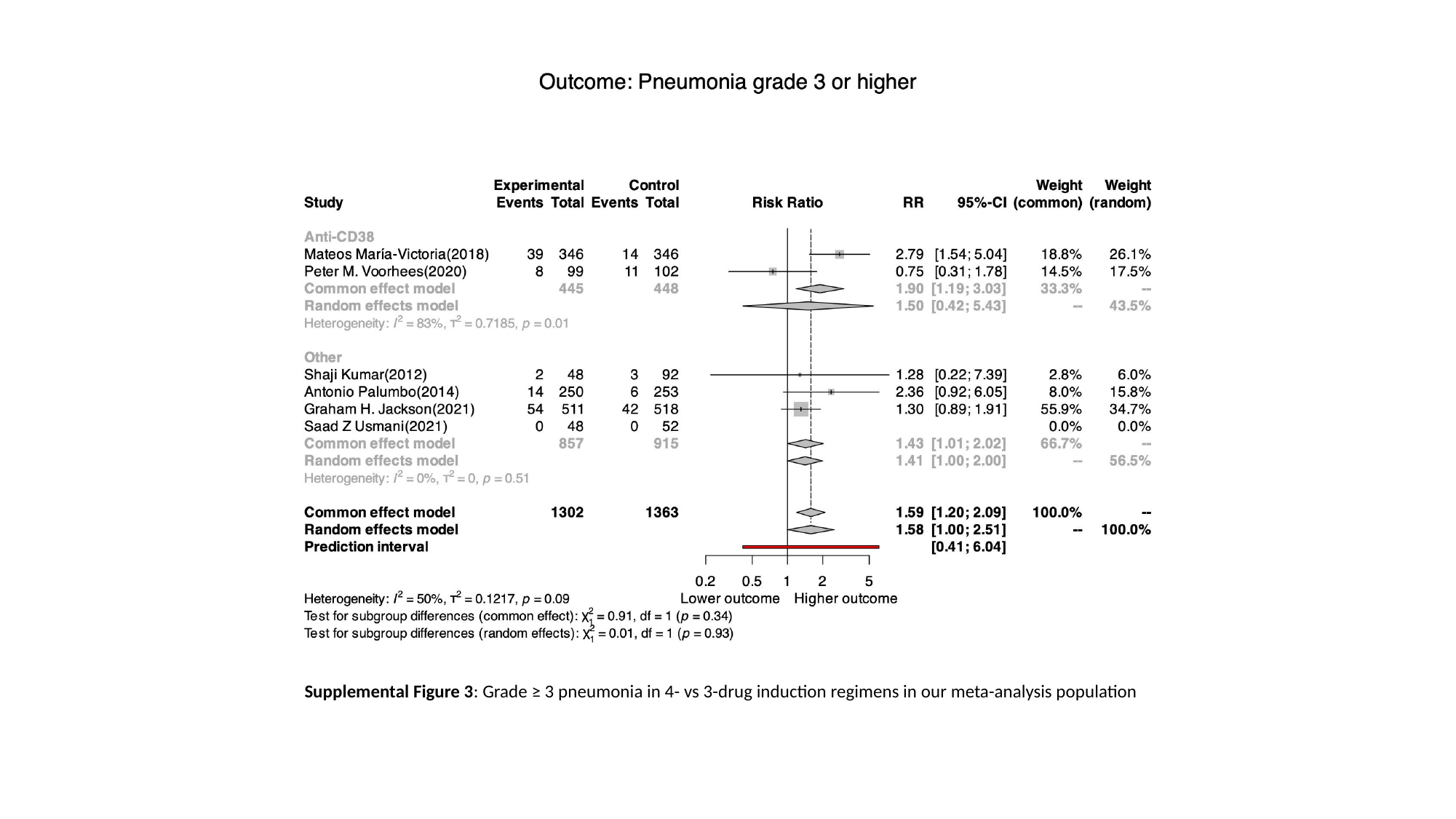

Supplemental Figure 3: Grade ≥ 3 pneumonia in 4- vs 3-drug induction regimens in our meta-analysis population
